# Comparative cytogenetics of two species of genus *Scobinancistrus* (Siluriformes, Loricariidae, Ancistrini) from the Xingu River, Brazil

**DOI:** 10.3897/CompCytogen.v7i1.4128

**Published:** 2013-03-18

**Authors:** Adauto Lima Cardoso, Karline Alves Holanda Sales, Cleusa Yoshiko Nagamachi, Julio Cesar Pieczarka, Renata Coelho Rodrigues Noronha

**Affiliations:** 1 Laboratório de Citogenética, Instituto de Ciências Biológicas, Universidade Federal do Pará – Campus do Guamá (Belém, PA, Brazil)

**Keywords:** Karyotypic divergence, chromosome rearrangements, sympatry

## Abstract

The family Loricariidae encompasses approximately 800 species distributed in six subfamilies. The subfamily Hypostominae consists of five tribes; of them, the tribe Ancistrini is relatively diverse, but it is not well known from the cytogenetic point of view. Genus *Scobinancistrus* Isbrücker et Nijssen, 1989, which is part of the tribe Ancistrini, has two species that occur in sympatry in the Xingu River, Brazil. In this work, we performed the first karyotypic characterizations of these two species and sought to identify the processes involved in their karyotypic evolution. Chromosomal preparations were subjected to Giemsa staining, silver nitrate impregnation, C-banding, CMA_3_ staining, DAPI staining, and FISH (fluorescence *in situ* hybridization) with 18S rDNA and telomeric probes. We found that *Scobinancistrus aureatus* Burgess, 1994 and *Scobinancistrus pariolispos* Isbrücker et Nijssen, 1989 shared the diploid number, 2n=52, but differed in their karyotypic formulae (KFs), distribution of constitutive heterochromatin (CH), and the localizations of their nucleolus organizer regions (NORs), which were found on the interstitial and distal regions of the long arm of chromosome pair 3 in *Scobinancistrus aureatus* and *Scobinancistrus pariolispos* respectively. We suggest that these interspecific variations may have arisen via paracentric inversion or transposition of the NOR. The karyotypic differences found between these two *Scobinancistrus* species can be used to identify them taxonomically, and may have functioned as a mechanism of post-zygotic reproductive isolation during the speciation process.

## Introduction

The fishes of the family Loricariidae are an important component of the ichthyofauna in the Neotropical region, where they are widely distributed and occupy a great variety of freshwater environments (Isbrücker 1980). The 800 known species are organized into six subfamilies: Hypoptopomatinae, Hypostominae, Lithogeninae, Loricariinae, Neoplecostominae and Delturinae (Armbruster 2004, Reis et al. 2006). The subfamily Hypostominae encompasses five tribes, Corymbophanini, Rhinelepini, Hypostomini, Ancistrini and Pterygoplichthyini, with the latter two forming the most derived clade (Armbruster 2004). The Ancistrini include numerous species with several yet-unsolved taxonomic problems, making it difficult to recognise them (Alves et al. 2003).

Cytogenetic information is incipient, given the great diversity of Ancistrini species, and has been efficient in distinguishing species of this tribe (Alves et al. 2003, Souza et al. 2009). It is therefore necessary to increase the amount of such information in order to improve the taxonomic identification of these fishes and understanding the evolutionary processes in this group. The members of the tribe Ancistrini have diploid numbers that vary from 2n=34 in *Ancistrus* sp. from the Purus river to 2n=52 in most of the other species karyotyped to date. Several sex chromosome systems have been identified, including simple and multiple systems, with either the male or the female as heterogametic sex. The numbers and localizations of the nucleolus organizer regions (NORs) are also rather variable; some species have simple NORs while others have multiple NORs (Artoni and Bertollo 2001, Alves et al. 2003, 2006, Souza et al. 2004, 2009, De Oliveira et al. 2006, 2007, 2008, 2009).

The genus *Scobinancistrus* Isbrücker et Nijssen, 1989 (Hypostominae, Ancistrini) comprises two species: *Scobinancistrus pariolispos* Isbrücker et Nijssen, 1989, which occurs in the Tapajós, Xingu and Tocantins Rivers, Brazil, and *Scobinancistrus aureatus* Burgess, 1994, which is endemic to the Xingu River (Fisch-Muller 2003, Camargo et al. 2004). They differ in that *Scobinancistrus pariolispos* has a fully evertible operculum, a final ray of the dorsal fin that extends as far as the adipose, and fins with no orange coloration (Camargo et al. 2012). No previous study has provided cytogenetic information for the two species of this genus.

In the present work, we obtained *Scobinancistrus pariolispos* and *Scobinancistrus aureatus* from the Xingu River and studied their karyotypes, in an effort to identify the processes involved in their karyotypic evolution and contribute new cytogenetic information for members of the tribe Ancistrini.

## Material and methods

**Figure 1.**
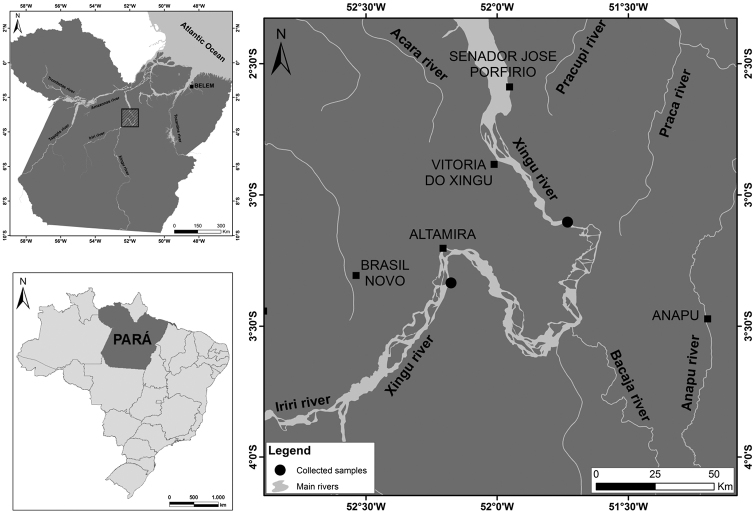
Collection localities of the analyzed *Scobinancistrus* samples.

Samples of *Scobinancistrus aureatus* (seven females) and *Scobinancistrus pariolispos* (five males and two females) from the Xingu River, Brazil, were analyzed (Fig. 1). Metaphase chromosomes were obtained according to the method described by Bertollo et al. (1978) and analyzed by conventional staining (Giemsa), C-banding (Sumner 1972), Ag-NOR labeling (Howell and Black 1980), chromomycin A_3_ staining (Schweizer 1980), DAPI staining (Pieczarka et al. 2006), and fluorescence *in situ* hybridization (FISH) with 18S ribosomal DNA (rDNA 18S) probes obtained from *Prochilodus argenteus* Agassiz, 1829 (Hatanaka and Galetti 2004) and human telomeric sequence probes (Oncor). The probes were labeled with biotin or digoxigenin by nick translation and detected with avidin-CY3 or anti-digoxigenin-FITC. The chromosomes were arranged according to the procedure described by Levan et al. (1964).

## Results

**Figure 2.**
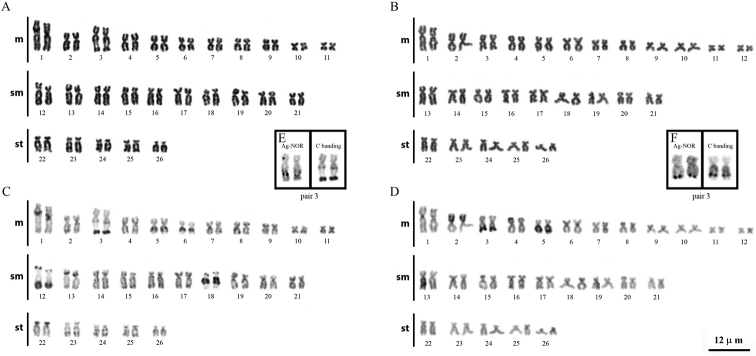
Karyotypic analyses of *Scobinancistrusaureatus* (**A, B** and **E**) and *Scobinancistrus pariolispos* (**B, D** and **F**): conventional staining (**A** and **B**), C-banding (**C** and **D**) and the NOR-bearing chromosome pair (**E** and **F**). The scale bar refers to all images.

The specimens of *Scobinancistrus aureatus* and *Scobinancistrus pariolispos* obtained from the Xingu River both had diploid numbers 2n=52 chromosomes, but they differed in their karyotypic formulas (KFs), which were 22m-20sm-10st and 24m-18sm-10st, respectively (Fig. 2A, B). Both males and females were analyzed for *Scobinancistrus pariolispos*, but no sex chromosomes were identified.

C-banding failed to identify constitutive heterochromatin (CH) in the centromeric region of any chromosome in the studied species. In *Scobinancistrus aureatus*, large heterochromatic blocks were seen, as follows: in the proximal regions of the long arms of chromosome pairs 5, 6 and 18; in the proximal regions of the short arms of pair 13; throughout the short arms of pair 22; in the distal regions of the long arms of pairs 3 and 12; and in the distal regions of the short arms of pairs 12 and 18 (Fig. 2C). In *Scobinancistrus pariolispos*, conspicuous heterochromatic blocks were also identified throughout the short arms of pair 2, in the distal regions of the long arms of pair 3, and throughout the long arms of pair 5 (Fig. 2D).

**Figure 3.**
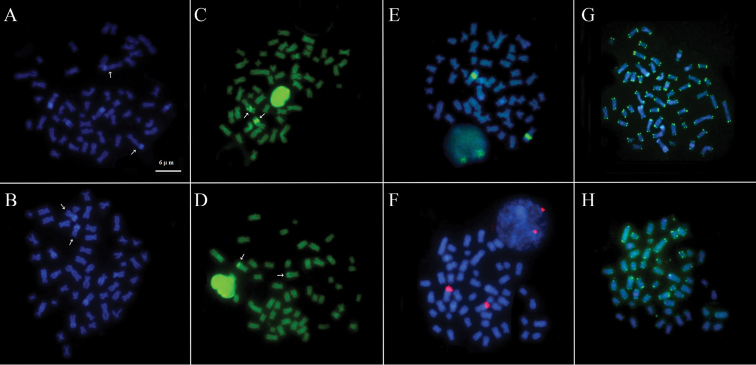
Further karyotypic analyses of *Scobinancistrus aureatus* (**A, C, E** and **G**) and *Scobinancistrus pariolispos* (**B, D, F** and **H**): DAPI staining (**A** and **B**), CMA_3_ staining (**C** and **D**), FISH with 18S rDNA probes (**E** and **F**) and FISH with telomeric sequence probes (**G** and **H**). The scale bar refers to all images.

A NOR was identified on a single chromosome pair per species. In *Scobinancistrus aureatus*, the NOR was located in the interstitial region of the long arms of pair 3, flanked by CH (Fig. 2E). In *Scobinancistrus pariolispos*, the NOR was situated in the distal region of the long arms of pair 3, adjacent to a block of CH (Fig. 2F). The numbers and localizations of these regions were confirmed by FISH with 18S ribosomal DNA probes (Fig. 3E, F).

The DAPI fluorochrome labeled the heterochromatic regions (Fig. 3A, B), and CMA_3_ stained the NORs (Fig. 3C, D). In FISH using telomeric sequence probes (TTAGGG), the probes hybridized to the ends of all chromosomes, but no interstitial telomeric labeling was observed (Fig. 3G, H).

## Discussion

*Scobinancitrus aureatus* and *Scobinancistrus pariolispos* from the Xingu River were found to have the same diploid number (2n=52), but their KFs differed. This is consistent with most other species of tribe Ancistrini, which share 2n=52 and differ in their KFs (Artoni and Bertollo 2001, Alves et al. 2003, 2006, Souza et al. 2004, 2009, De Oliveira et al. 2007). Chromosomal inversions, which are rearrangements that can modify the structure of chromosomes without altering their number, may explain how these species have the same diploid number but different KFs (Alves et al. 2003, De Oliveira et al. 2006). According to Artoni and Bertollo (2001) and Kavalco et al. (2005), 2n=54 probably corresponds to a basal condition for the Loricariidae. Therefore, the reduction to 2n=52 in most of the Ancistrini species must be the result of a fusion event. The same type of rearrangement is believed to explain the reduction of the diploid number in several species of the genus *Ancistrus* Kner, 1854 (Alves et al. 2003, De Oliveira et al. 2009).

**Figure 4.**
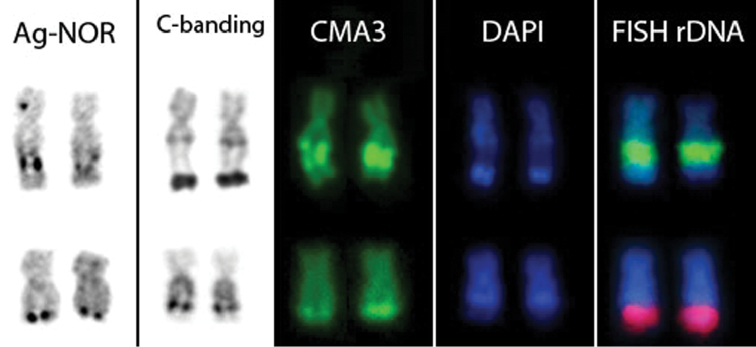
NOR-bearing chromosomes of *Scobinancistrus aureatus* (upper row) and *Scobinancistrus pariolispos* (lower row).

The NOR was found on the same chromosome pair in the two *Scobinancistrus* species studied here in this situation is shared by most members of the tribe Ancistrini (Artoni and Bertollo 2001, Alves et al. 2003, 2006, De Oliveira et al. 2007, Souza et al. 2004, 2009). The long arm of chromosome pair 3 was identified as the bearer of the NOR in both species; however, it was found in an interstitial, CH-flanked region in *Scobinancistrus aureatus*, but in the distal region, adjacent to a CH block, in *Scobinancistrus pariolispos*. This organization indicates that the NOR-bearing chromosomes are not the same in *Scobinancistrus aureatus* and *Scobinancistrus pariolispos*, suggesting the occurrence of events that changed the position of the NOR within the karyotype (Fig. 4). The repetitive nature of the ribosomal DNA that constitutes the NOR and its association with CH (which consists mainly of satellite DNA and transposable elements; Dimitri et al. 2009) may facilitate transposition events that can move the NOR to another region of the genome (Gross et al. 2009, 2010). It is important to point out that the determination of chromosome pair numbers in these species is tentative, and therefore pair 3 of one species is not necessarily homologous to pair 3 of another species. However, we cannot exclude the possibility that these pairs are homologous and reflect the occurrence of a paracentric inversion involving the NOR and nearby HC. The NORs labeled positive for the fluorochrome, CMA_3_, but negative for DAPI, indicating that the ribosomal DNA is interspersed with repetitive GC-rich DNA, as has been frequently described in other fishes (Pendás et al. 1993).

The constitutive heterochromatin stained positive with DAPI, indicating that it is AT-rich. Its patterns differed between the two species, with large heterochromatic blocks in non-centromeric regions located predominantly in non-homologous chromosomes, indicating that processes related to the dynamics of repetitive DNA (e.g., transposition) may have been involved in the karyotypic differentiation of these two species.

Using information on karyotype macrostructures and CH and NOR distribution, Souza et al. (2009) identified chromosomes with possible homologies among species of genus *Peckoltia* Miranda Ribeiro, 1912, which belong to the tribe Ancistrini. However, using same criteria, we were unable to identify any homologies between the karyotypes of *Scobinancistrus aureatus* and *Scobinancistrus pariolispos*. This suggests that the studied karyotypes may have undergone both inversions (as noted above) and reciprocal translocation events, leading to greater genomic reorganization. Consistent with this, Nagamachi et al. (2010) used chromosome painting to demonstrate that the differences between two *Gymnotus carapo* Linnaeus, 1758 populations involved a greater number of chromosome rearrangements than previously assumed based on classical cytogenetic data (Milhomem et al. 2008).

In conclusion, the karyotypic differences found in the two *Scobinancistrus* species studied herein can be used in their taxonomic identification. Moreover, the sympatric occurrence of these species suggests that the identified karyotypic differences may have functioned as a mechanism of post-zygotic reproductive isolation during the speciation process.
